# Comparison of DNA quantification methodology used in the DNA extraction protocol for the UK Biobank cohort

**DOI:** 10.1186/s12864-016-3391-x

**Published:** 2017-01-05

**Authors:** Samantha Welsh, Tim Peakman, Simon Sheard, Rachael Almond

**Affiliations:** UK Biobank, Units 1-4, Spectrum Way, Adswood, Stockport, SK3 0SA Cheshire UK

**Keywords:** UK Biobank, Genotyping, Affymetrix, DNA concentration, PicoGreen, Trinean, UV/Vis, Quantification

## Abstract

**Background:**

UK Biobank is a large prospective cohort study in the UK established by the Medical Research Council (MRC) and the Wellcome Trust to enable approved researchers to investigate the role of genetic factors, environmental exposures and lifestyle in the causes of major diseases of late and middle age. A wide range of phenotypic data has been collected at recruitment and has recently been enhanced by the UK Biobank Genotyping Project. All UK Biobank participants (500,000) have been genotyped on either the UK Biobank Axiom® Array or the Affymetrix UK BiLEVE Axiom® Array and the workflow for preparing samples for genotyping is described. The genetic data is hoped to provide further insight into the genetics of disease. All data, including the genetic data, is available for access to approved researchers.

Data for two methods of DNA quantification (ultraviolet-visible spectroscopy [UV/Vis]) measured on the Trinean DropSense™ 96 and PicoGreen®) were compared by two laboratories (UK Biobank and Affymetrix).

**Results:**

The sample processing workflow established at UK Biobank, for genotyping on the custom Affymetrix Axiom® array, resulted in high quality DNA (average DNA concentration 38.13 ng/μL, average 260/280 absorbance 1.91). The DNA generated high quality genotype data (average call rate 99.48% and pass rate 99.45%). The DNA concentration measured on the Trinean DropSense™ 96 at UK Biobank correlated well with DNA concentration measured by PicoGreen® at Affymetrix (r = 0.85).

**Conclusions:**

The UK Biobank Genotyping Project demonstrated that the high throughput DNA extraction protocol described generates high quality DNA suitable for genotyping on the Affymetrix Axiom array. The correlation between DNA concentration derived from UV/Vis and PicoGreen® quantification methods suggests, in large-scale genetic studies involving two laboratories, it may be possible to remove the DNA quantification step in one laboratory without affecting downstream analyses. This would result in reductions in cost and time to complete the project, allowing generation of genetic data faster and cheaper.

**Electronic supplementary material:**

The online version of this article (doi:10.1186/s12864-016-3391-x) contains supplementary material, which is available to authorized users.

## Background

UK Biobank is a resource containing samples and a wide range of data on 500,000 UK participants. The resource is available for approved researchers to apply to use for the purposes of ‘improving health of future generations’ [1–5].

An enhancement project was initiated in 2013 which aimed to genotype the entire UK Biobank cohort using a high density array and subsequent imputation. The majority of participants (~440,000) were genotyped on the UK Biobank Axiom® Array [6], with 50,000 participants genotyped on the Affymetrix UK BiLEVE Axiom® Array [7], which has > 95% content overlap with the UK Biobank Axiom® Array.

A bespoke sample processing workflow was designed to ensure the ~500,000 samples could be processed to a high quality within 18 months. The pipeline involved three main stages (and three collaborating entities); preparation of the DNA was performed at UK Biobank, genotyping at Affymetrix and quality control (QC) of the data at the Wellcome Trust Centre for Human Genetics (WTCHG). The Clinical Trial Service Unit (CTSU) at the University of Oxford is responsible for the storage and distribution of the genotype data (and all other UK Biobank data) to approved researchers.

It is well reported that the concentration obtained from DNA quantification can differ significantly dependent upon the DNA quantification method used, DNA extraction methodology and laboratory [8–11]. Since DNA was extracted at UK Biobank and genotyped at Affymetrix (following normalisation), both laboratories included a method to quantify the DNA for QC purposes. This is typical of assays where the DNA sample is not extracted in-house.

UK Biobank used the Trinean DropSense™ 96 droplet reader in combination with DropPlate-S (a UV/Vis-based plate method) and Affymetrix used PicoGreen® (a fluorescent dye that intercalates with DNA). This project provided a unique opportunity to compare two commonly used DNA quantification methods across a large sample number. A comparison of these two DNA quantification methods across two laboratories is presented.

## Methods

Methods below describe the DNA extraction/genotyping workflow for the ~440,000 UK Biobank samples run on the UK Biobank Axiom® Array. Further detail on the 50,000 UK Biobank samples run on the UK BiLEVE Axiom® Array is available elsewhere [7].

## UK Biobank

### Selection of source sample for DNA extraction

UK Biobank participants provide a wide range of biological samples [4, 5] that are aliquotted into 850 μL, 2D bar-coded micro tubes prior to storage. The buffy coat aliquot, derived from 10 ml of whole blood collected into an ethylenediaminetetraacetic acid (EDTA) vacutainer, was selected for DNA extraction as it was available for the majority of participants and was expected to yield the required 10 ng/μL concentration required for genotyping. Saliva was considered but due to the lower number of participants providing this sample type to the UK Biobank study, this was not used.

During retrieval of buffy coat samples for DNA extraction it was important to maximise the picking speed whilst avoiding clustering of participants by time or date of collection, collection centre, geography (UK Biobank recruited from 22 assessment centres across the UK), or any participant phenotype (typically only 2 assessment centres were represented on each stored plate). An algorithm was developed to pick the samples in a way that increased the number of assessment centres per plate to reduce potential systematic bias from the way the samples were originally collected and processed that could affect downstream genotyping.

### DNA extraction

**Fig. 1:**
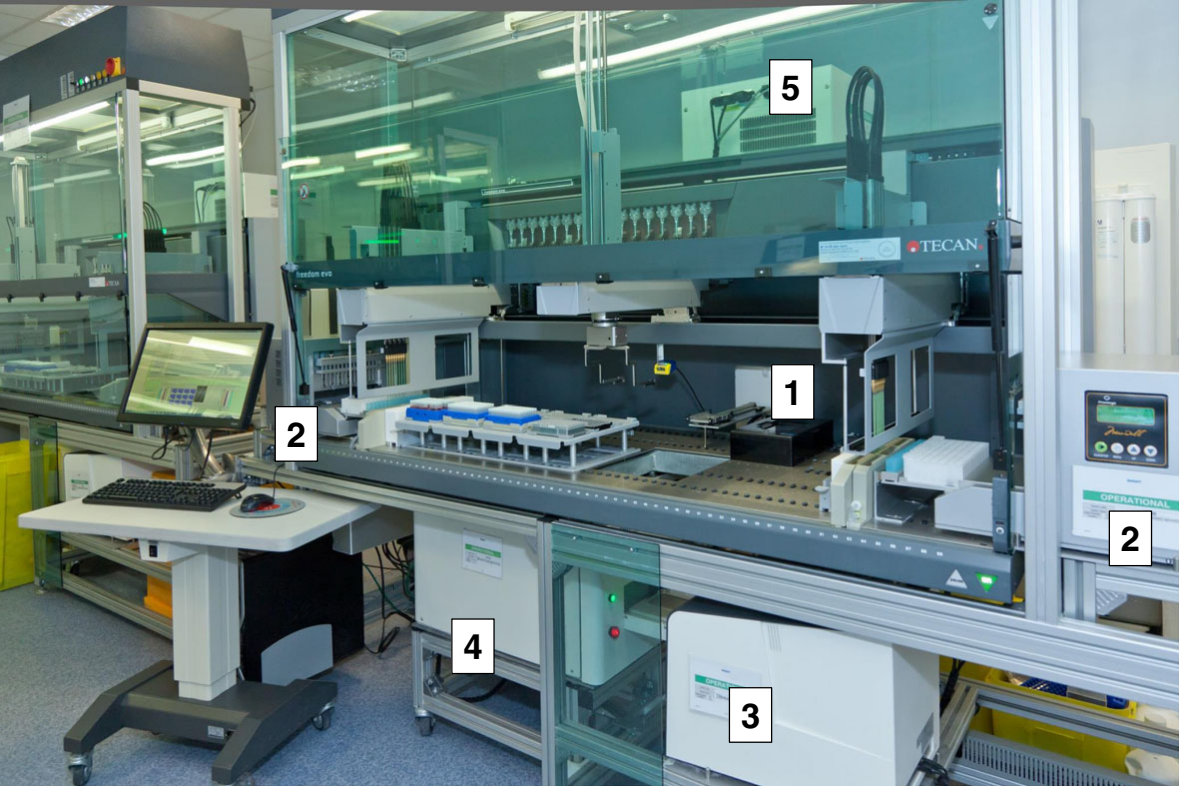
UK Biobank DNA Extraction System. This system comprises 1) Ziath® DataPaq High Speed Single Rack Scanner ZTS-A6 for sample tracking, 2) two modified hemi-skinned Promega Maxwell® 16 instruments for DNA extraction, 3) Trinean DropSense™ 96 for DNA quantification, 4) Brooks Tube Auditor™ for volume measurement and 5) Liconic STX44 Automated Incubator to keep DNA at 4 °C after extraction

A custom DNA extraction system was developed by the Tecan Integration Group (TIG) [12] to enable DNA extraction and quantification in a single process (Fig. 1). DNA was extracted from 850 μL of buffy coat using a cartridge-based, magnetic bead extraction methodology (Maxwell® 16 Blood DNA Purification Kit, Promega, AS1010X and Maxwell® 16 Research Instrument [Promega, AS200-HS]).

DNA extraction occurs within the pre-filled cartridge (supplied in Promega, AS1010X) which is split into wells containing lysis buffer, MagneSil™ Paramagnetic Particles (PMPs) and wash buffers. Briefly, DNA is moved between wells by the PMPs and the application of a magnetic force to disposable plungers. Cells are lysed by a guanidine-based lysis buffer alongside mechanical lysis from the disposable plungers. DNA adsorbed by silica coated magnetic beads is moved through a series of wash wells by a plunger. Salts from lysis and other impurities that may inhibit downstream processing (e.g. haem, proteins etc.) are removed during the washing. Once purified, DNA is eluted from the beads in Tris-EDTA-based lysis buffer, is assisted by heating at 56 °C.

To maximise the DNA yield and purity from the source material (standard protocol extracts from 250 μL buffy coat), the lysis buffer (Promega, A826E) was increased by 600 μL, the wash buffer (Promega, MD1412) by 1 mL in two wash wells and the cycle through the cartridge was repeated a further two times.

Following extraction, the DNA was aliquotted across three tubes; one for primary storage at UK Biobank (425 μL), one for back-up storage at UK Biobank back-up centre (425 μL) and one for genotyping (50 μL). The primary aliquot was quantified on the Trinean DropSense™ 96.

The quality metrics for a 96-well plate to automatically proceed to genotyping on the Axiom® array was 80% of the plate must have a DNA concentration > 10 ng/μL. Prior to shipping a plate for genotyping, the measured DNA concentration and quality (by absorbance at 260/280) of the stock DNA (sibling to the DNA shipped for genotyping) were assessed from the concentration obtained from the Trinean DropSense™ 96. Results from QC checks were entered into the UK Biobank Laboratory Information Management System (LIMS) and are a data field that can be requested by researchers.

### Shipment

Following DNA extraction and quantification, DNA was stored at −80 °C. Plates were sent for genotyping to the Affymetrix Research Services Lab, Santa Clara, CA, USA (ARSL) approximately weekly. Plates were shipped on dry ice and accompanied by an electronic sample manifest containing an anonymised participant identifier plus the gender, ethnicity and geographical location of the participant to which the sample pertains (latter for QC purposes).

### Pre-genotyping at affymetrix

Genotyping was performed at the ARSL as per the Manufacturer’s Instructions [13] using the UK Biobank Axiom® Array or UK BiLEVE Axiom® Array.

In summary, samples were thawed and homogenised (incubated at 37 °C for 2 h) prior to PicoGreen® quantification to establish the volume of DNA required for normalisation to a concentration of 10 ng/μL. Plates where < 80% of samples had a concentration of > 10 ng/μL required authorisation before proceeding to genotyping. After normalisation, two controls were added to the plate and samples entered the Axiom® assay workflow [14]. Samples failing initial QC (<95% of markers measured could be confidently genotyped [call rate]) were re-processed. If samples failed re-processing a second sample from the participant was extracted, where available.

Data analysis was performed as per Manufacturer’s Guidelines [15]. Any deviations from standard protocol are documented in [14].

### Data QC at WTCHG

QC procedures applied to the UK Biobank genotyped data are described in [16]. Upon completion of QC, data is passed to UK Biobank for release to approved researchers.

## Results and discussion

The sample processing workflow described aimed to generate high quality genotypes from as many UK Biobank participants as possible. At UK Biobank, it was important to design a workflow that generated high quality DNA for genotyping on the Axiom^®^ Array (and use in future downstream applications), whilst maintaining a high throughput so data were available within a short timeframe. Results are presented for 484,325 samples. Approximately 1% of samples (5016 samples) were excluded from the analysis because DNA quantification data was not available from both Trinean and PicoGreen quantification.

### Success of picking algorithm

**Fig. 2:**
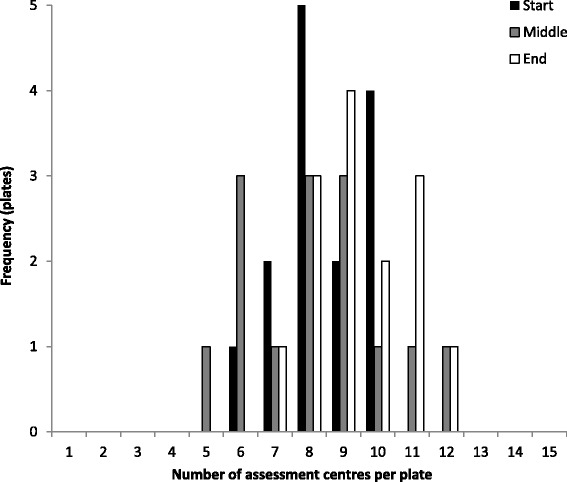
Assessment centres per picked plate. Number of assessments centres per picked plate from three sets of 14 plates from time-points during picking (*start*, *middle* and *end*)

The picking algorithm was designed to prevent clustering of phenotypes (specifically participant assessment centre). The success of the algorithm was assessed by selecting 14 plates picked consecutively at the start, middle and end of the project and counting the number of assessment centres represented on each plate. Of the 42 plates checked, each plate contained samples from participants from at least five assessment centres (Fig. 2). This demonstrates the picking algorithm was successful in avoiding clustering of samples by assessment centre and reducing sample bias on the genotyping plate.

### Success of DNA extraction—DNA quantity and quality assessment at UK Biobank

**Fig. 3:**
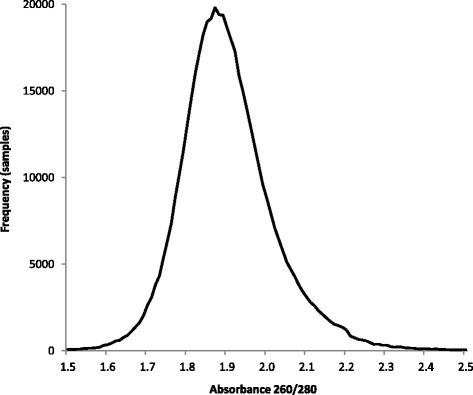
DNA quality assessed by 260/280. Spread of 260/280 values across 481,772 DNA samples (results from 2553 samples outside 1.5–2.5 not displayed)

The novel methodology to extract DNA from 850 μL of buffy coat was assessed via the DNA concentration and 260/280, using the Trinean DropSense™ 96 (a UV/Vis, plate-based DNA quantification system). The average DNA concentration was 38.13 ng/μL (0.02 to 634.99 ng/μL) and average 260/280 was 1.91 (distribution shown in Fig. 3). The results from quantification at UK Biobank indicated the DNA extraction protocol generated good quality DNA. From DNA quantification performed at UK Biobank, 1.31% of samples fell outside the required 10 ng/μL threshold for automatic pass into the genotyping process.

### DNA quantification—comparison of methods

This project provided a unique opportunity to compare two methods of DNA quantification across a large number of samples. DNA concentration measured via the UV/Vis on the Trinean DropSense™ 96 during DNA extraction at UK Biobank were exported alongside the PicoGreen® derived concentrations included in the summary metrics from Affymetrix for each sample from the UK Biobank LIMS.

**Fig. 4:**
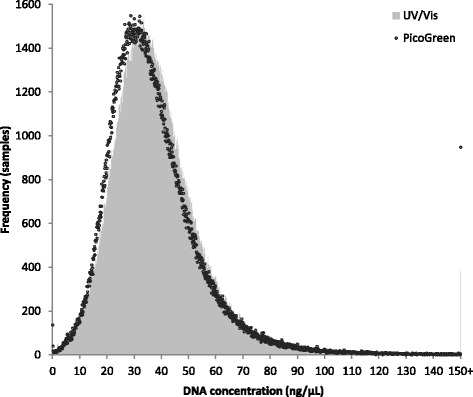
DNA concentration measured by UV/Vis (Trinean) and PicoGreen®. Spread of DNA concentration in 484,325 samples quantified using Trinean DropSense™ 96 and PicoGreen®

Out of the 484,325 samples where data were available from both quantification methods, the average DNA concentration measured using the Trinean DropSense™ 96 was 38.13 ng/μL (0.02 to 634.99 ng/μl) and via PicoGreen® was 37.13 ng/μL (0.01 to 730.52 ng/μL; Fig. 4).

**Fig. 5:**
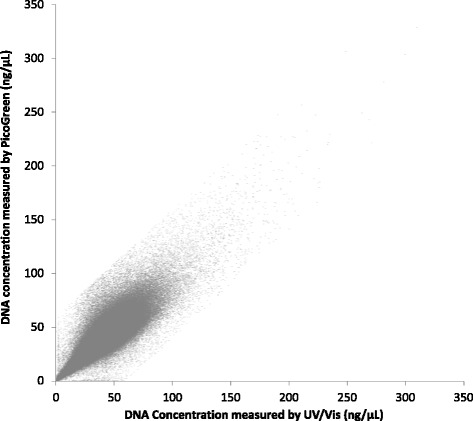
Correlation between UV/Vis (Trinean) and PicoGreen® methods of DNA quantification. Correlation between DNA concentration measured via Trinean DropSense™ 96 and PicoGreen® quantification methods in 482,638 samples (r = 0.85)

Correlation of the two methods was assessed between the DNA concentration obtained from Trinean DropSense™ 96 and PicoGreen® quantification methods from 482,638 samples (r = 0.85; Fig. 5; Additional file 1). Some samples were excluded from the analysis (1687 samples; 0.3%) as there was > 60 ng/μL difference between the DNA concentration measured by UV/Vis and PicoGreen quantification methods. This difference in a small number of samples can be explained by the lack of homogeneity in some of the DNA samples. The DNA samples were quantified by UV/Vis immediately after extraction; it is expected that the DNA is largely homogenous at this stage as it has recently been eluted using a combination of mechanical mixing with a plunger and heat (with the exception of highly viscous samples). Conversely, PicoGreen® quantification was performed after storage at −80 °C, shipping on dry ice and homogenisation by heating. It is possible that a small number of viscous samples were heterogeneous during one or both of the DNA quantifications resulting in large differences in the measured DNA concentrations [17].

Additionally, there were a small number of samples where one method quantified the DNA near 0 ng/μL and the other method quantified DNA > 10 ng/μL (87 samples were quantified as < 2 ng/μL via UV/Vis and > 10 ng/μL via PicoGreen® and 233 samples were quantified as < 2 ng/μL via PicoGreen® and > 10 ng/μL via UV/Vis [Additional file 2]). The most likely explanation for the difference would be failure to transfer DNA to the quantification plate in one of the quantification methods during automation.

The correlation demonstrates that whilst others have observed differences between DNA concentration methodologies [10, 11], the preparation of DNA as described in this paper yields similar DNA concentrations whether measured using a UV/Vis method (on the Trinean DropSense™ 96) or via PicoGreen®. The findings from this comparison may assist other laboratories when considering the sample workflow and whether repeat DNA quantifications of the same sample are required.

### Success of genotyping on axiom® array

Results from DNA concentration checks at UK Biobank indicated that the majority of samples would meet the requirements for genotyping of DNA concentration > 10 ng/μL and 260/280 > 1.8 (98.7% of samples > 10 ng/μL, 84.3% 260/280 > 1.8). Results from genotyping (after QC by Affymetrix, prior to WTCHG QC) for 484,325 samples (average call rate 99.48% and pass rate 99.45%) demonstrate the DNA generated from the workflow described is of high quality and is suitable for downstream genetic analysis; Axiom genotyping in this case.

The average DNA concentration of the 2675 failed samples was 36.54 ng/μL (UV/Vis) and 37.15 ng/μL (PicoGreen®) compared with 38.14 ng/μL and 34.71 ng/μL (UV/Vis and PicoGreen®, respectively) for samples that passed. The average 260/280 of failed samples was 1.93 and 1.91 for samples that passed. DNA concentration metrics from pass and fail samples do not demonstrate that DNA quantity or quality was not responsible for the small percentage of failures at the genotyping step.

## Conclusions

The UK Biobank Genotyping project was established to generate genotype data from as many of the 500,000 participants as possible. Data from 150,000 participants is already available to approved UK Biobank researchers and will be followed in 2016 by the data from the remaining participants upon completion of QC and imputation by WTCHG.

The sample processing workflow described has generated high quality DNA for the UK Biobank Genotyping Project and for use in future UK Biobank projects. The comparison between DNA quantification has demonstrated Trinean DropSense™ 96 quantification performed at UK Biobank and PicoGreen® performed at Affymetrix generates similar results (r = 0.85) which may be improved further by thorough optimisation of the DNA homogenisation protocol following thawing. The correlation between DNA concentration derived from UV/Vis and PicoGreen® quantification methods in this workflow suggests, in large-scale genetic studies involving two or more laboratories, it may be possible to remove repeat DNA quantification steps without affecting downstream analyses. It is recommended that during the early phases of a project, the correlation between quantification methods employed in each laboratory is assessed with the aim of removing one of the quantification measures if the correlation is good. Removing a quantification step in the workflow would lead to increased throughput, a decrease in consumable costs and a reduction in staff required which would ultimately allow for generation of genetic data faster and cheaper.

## Additional files

Additional file 1: Correlation between UV/Vis (Trinean) and PicoGreen® methods of DNA quantification (r = 0.85). Solid line r = 1; dashed line = correlation UV/Vis and PicoGreen® quantification methods across 482,638 samples (r = 0.85). (PDF 1692 kb)
Additional file 2: Correlation between UV/Vis (Trinean) and PicoGreen® methods of DNA quantification for DNA concentration < 60 ng/μL. Correlation plot showing all data points (442,859 samples) where DNA concentration measured via UV/Vis is < 60 ng/μL. Samples quantified via one method < 2 ng/ μL and with another method > 10 ng/μL are highlighted with a black cross. (PDF 166 kb)

## Abbreviations

ARSL: Affymetrix Research Service Laboratory, Santa Clara, CA, USA
CTSU: Clinical Trials Service Unit
EDTA: Ethylenediaminetetraacetic acid
LIMS: Laboratory Information Management System
MRC: Medical Research Council
PMPs: Paramagnetic particles
QC: Quality control
TIG: TECAN Integration Group
UK BiLEVE: UK Biobank Lung Exome Variant Evaluation
UV/Vis: Ultraviolet/visible spectroscopy
WTCHG: Wellcome Trust Centre for Human Genetics
