# Supplementary figures and images for: Comparison of DNA quantification methodology used in the DNA extraction protocol for the UK Biobank cohort
Source: BMC Genomics. 2017 Jan 5;18:26. doi: 10.1186/s12864-016-3391-x (PMC5217214; doi:10.1186/s12864-016-3391-x)

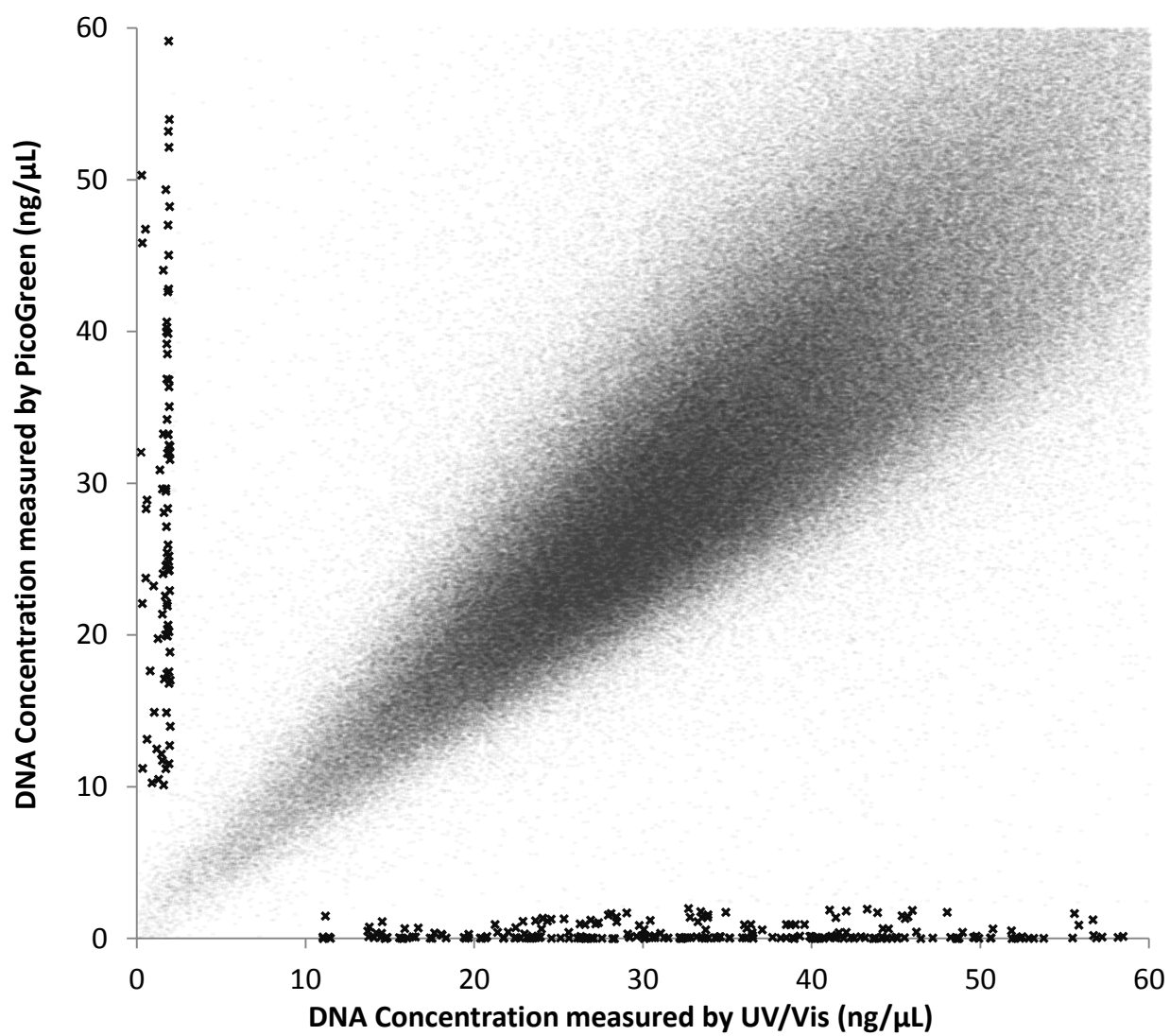

Supplement: Additional file 1: — Correlation between UV/Vis (Trinean) and PicoGreen® methods of DNA quantification (r = 0.85). Solid line r = 1; dashed line = correlation UV/Vis and PicoGreen® quantification methods across 482,638 samples (r = 0.85). (PDF 1692 kb) [file 12864_2016_3391_MOESM1_ESM.pdf]

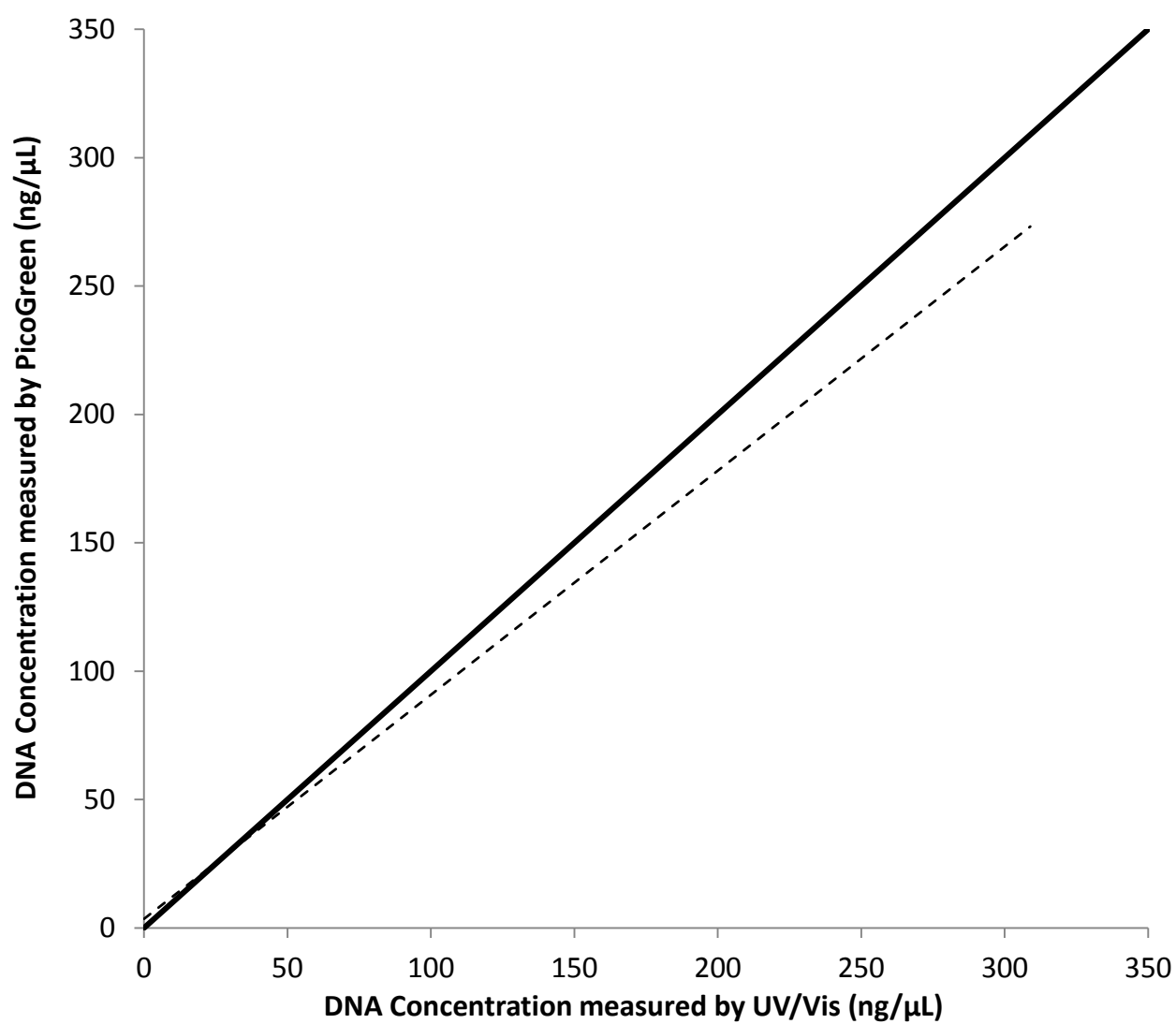

Supplement: Additional file 2: — Correlation between UV/Vis (Trinean) and PicoGreen® methods of DNA quantification for DNA concentration < 60 ng/μL. Correlation plot showing all data points (442,859 samples) where DNA concentration measured via UV/Vis is < 60 ng/μL. Samples quantified via one method < 2 ng/ μL and with another method > 10 ng/μL are highlighted with a black cross. (PDF 166 kb) [file 12864_2016_3391_MOESM2_ESM.pdf]
